# Supplementary figures and images for: Comparative analysis of two Caenorhabditis elegans kinesins KLP-6 and UNC-104 reveals a common and distinct activation mechanism in kinesin-3
Source: eLife. 2024 Jan 11;12:RP89040. doi: 10.7554/eLife.89040 (PMC10945585; doi:10.7554/eLife.89040)

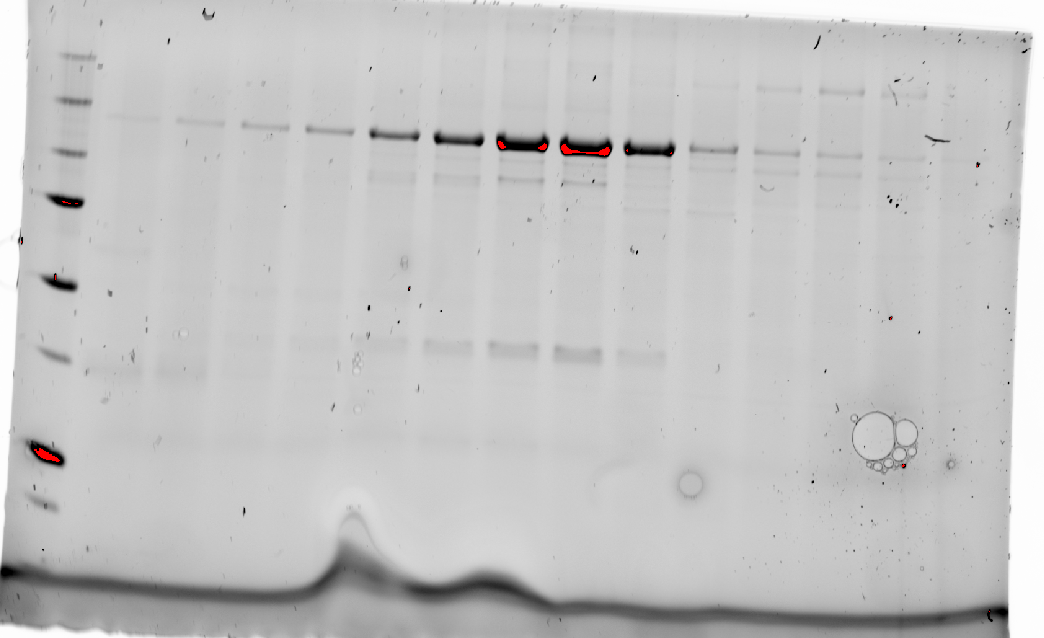

Supplement: Figure 1—source data 1. [file elife-89040-fig1-data1.zip › Figure 1ΓÇösource data 1/KLP-6FL.tif]

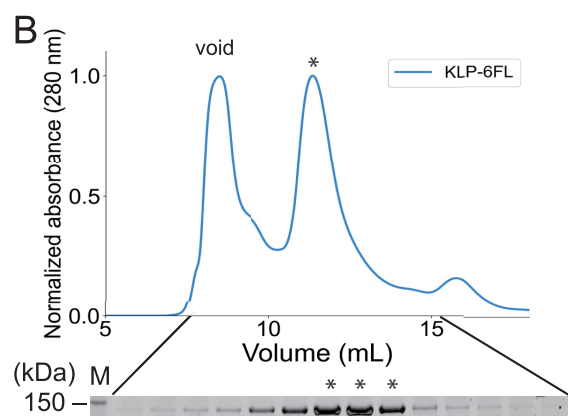

KLP-6FL

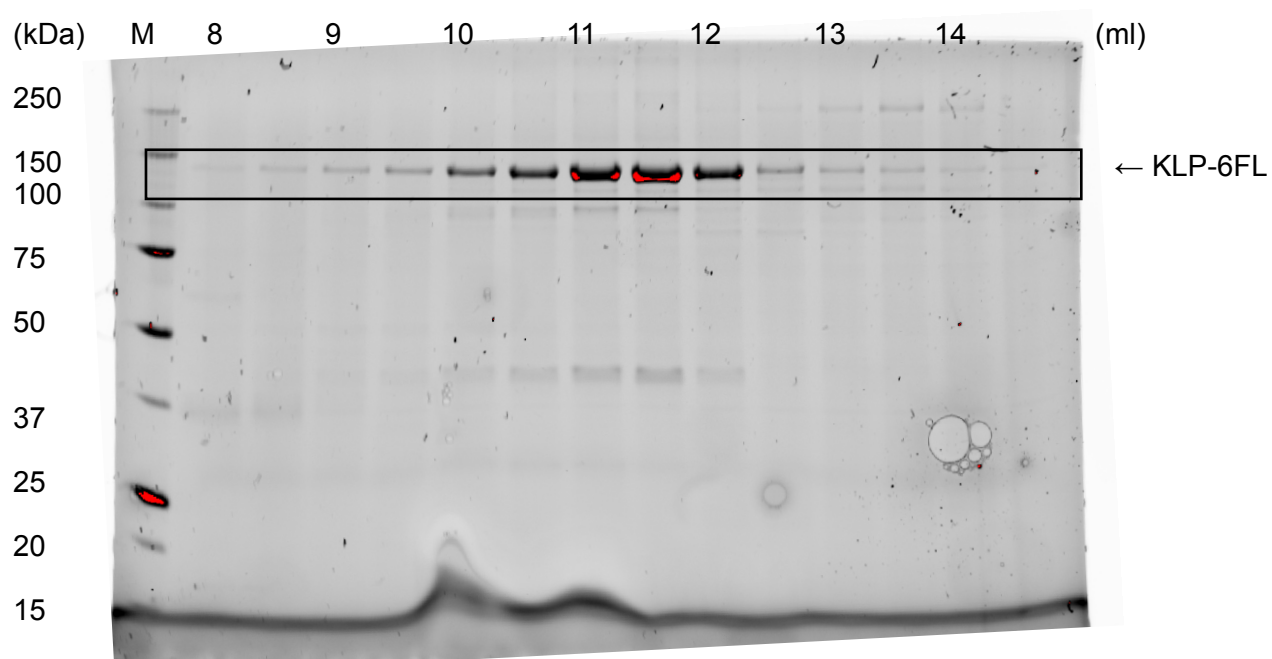

Supplement: Figure 1—source data 2. [file elife-89040-fig1-data2.zip › Figure 1ΓÇösource data 2/Figure 1ΓÇösource data 2.pdf]

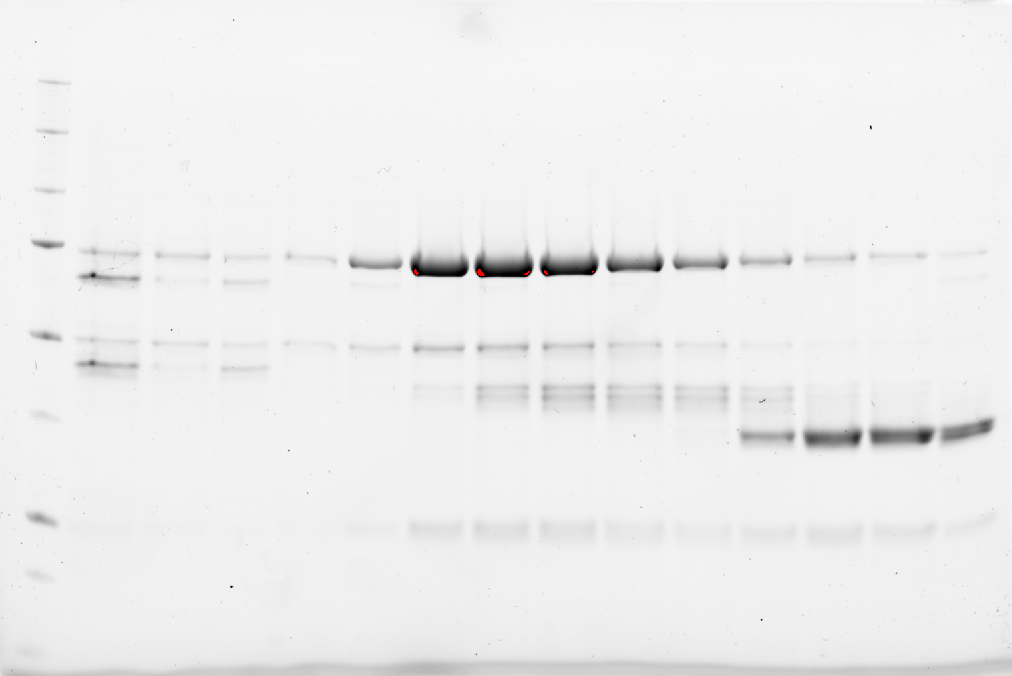

Supplement: Figure 3—source data 2. [file elife-89040-fig3-data2.zip › Figure 3ΓÇösource data 2/KLP-6(1-390)LZ.tif]

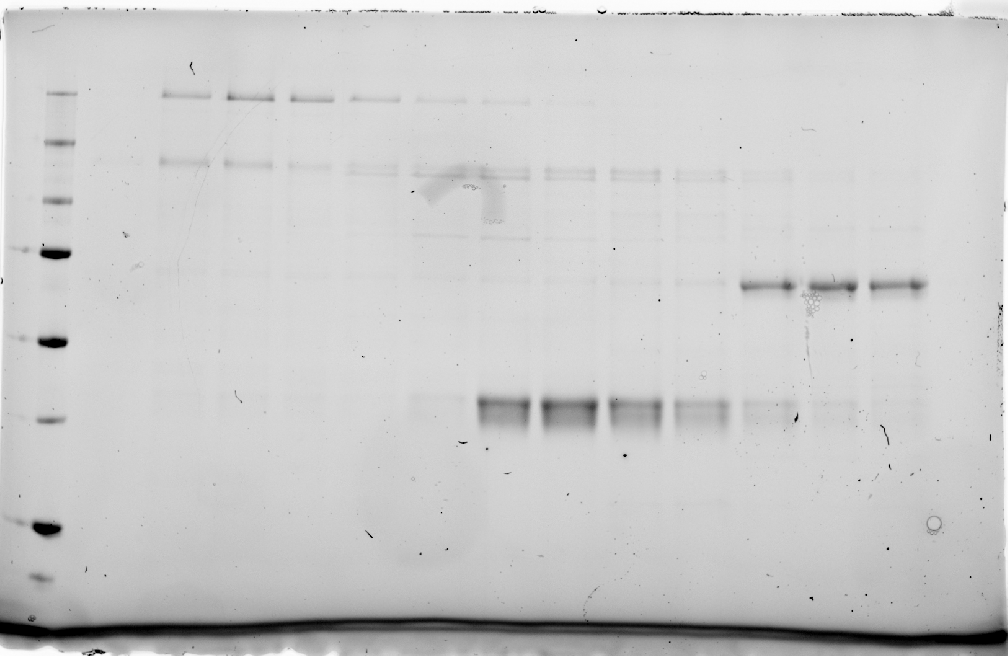

Supplement: Figure 3—source data 2. [file elife-89040-fig3-data2.zip › Figure 3ΓÇösource data 2/KLP-6(1-390).tif]

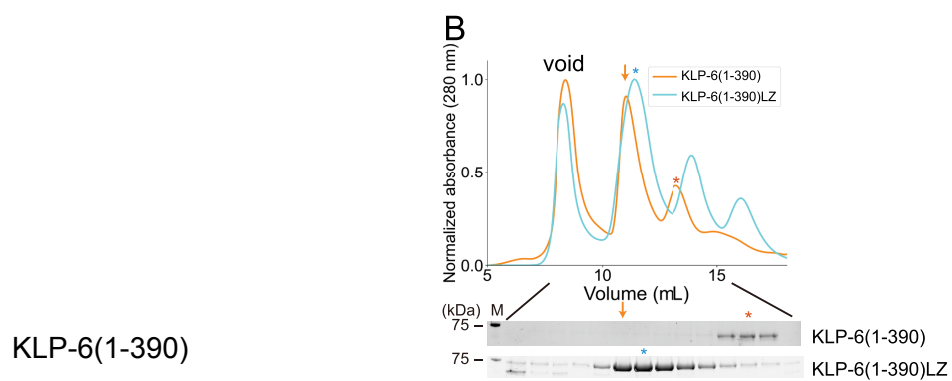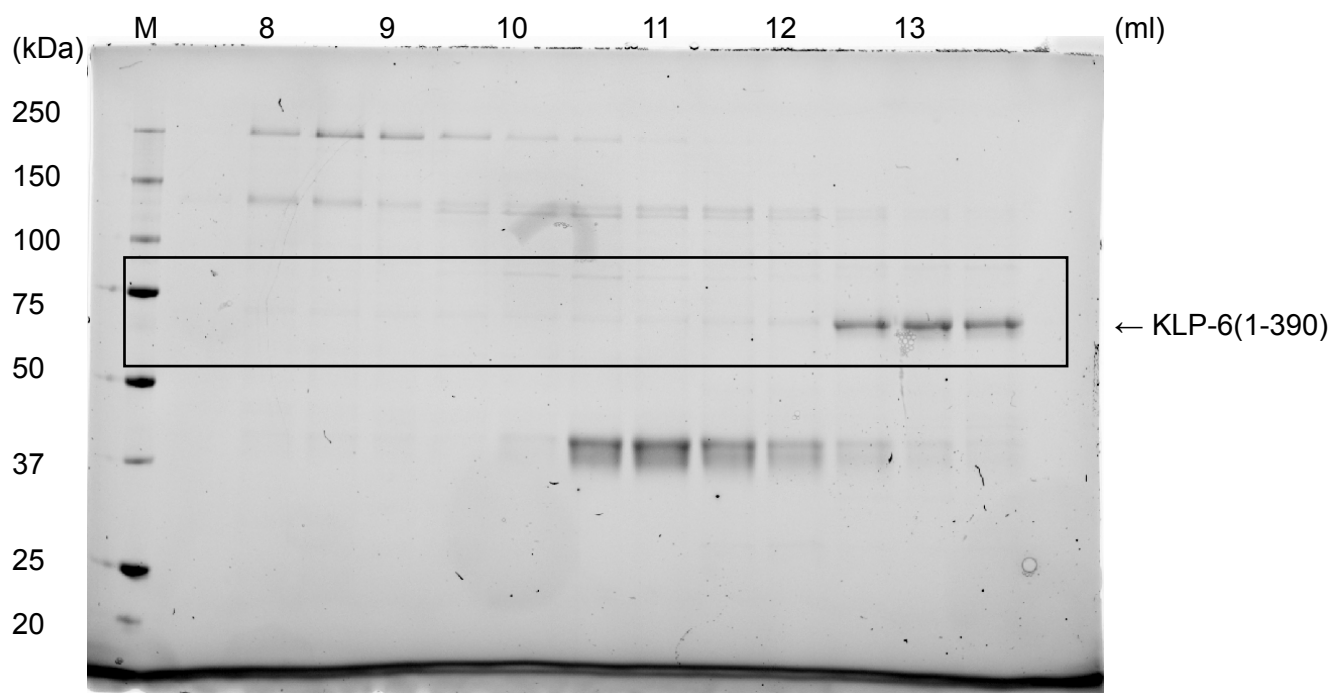

KLP-6(1-390)LZ

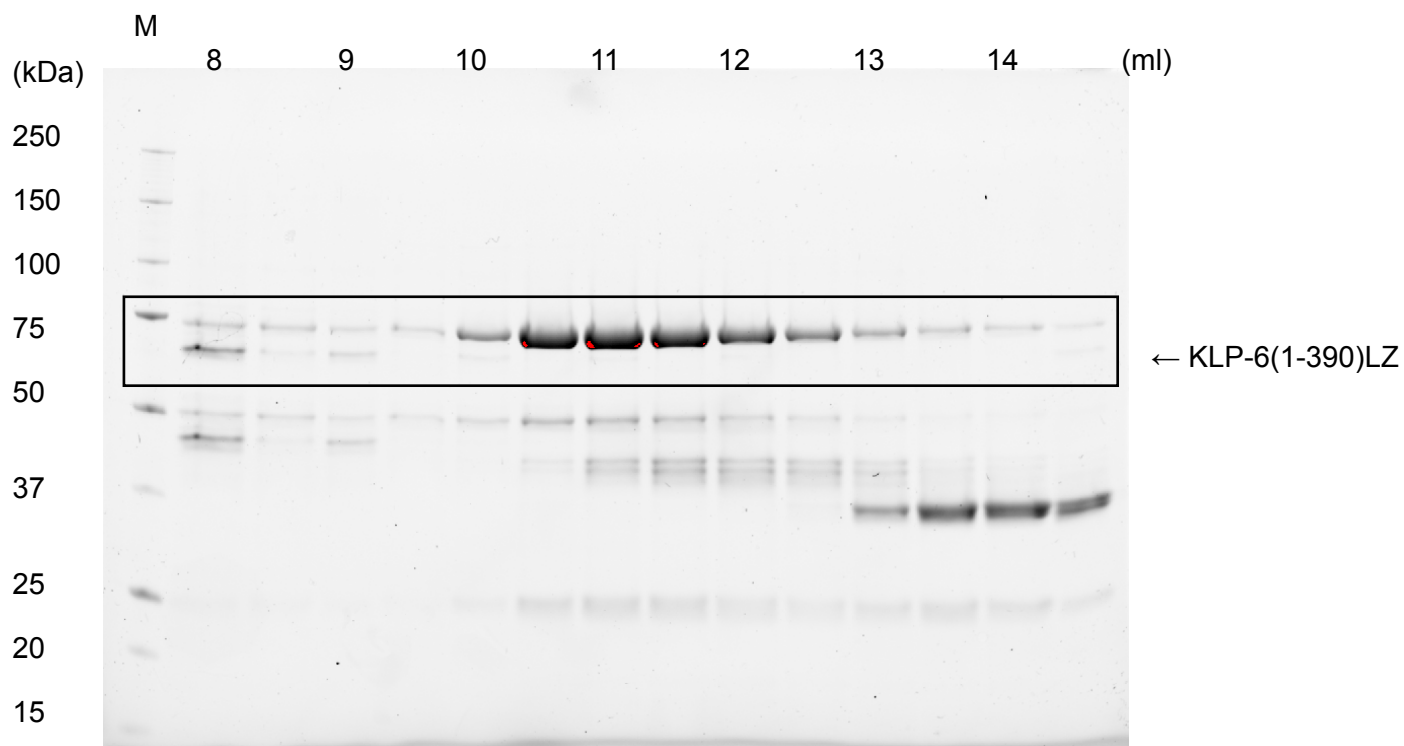

Supplement: Figure 3—source data 3. [file elife-89040-fig3-data3.zip › Figure 3ΓÇösource data 3/Figure 3ΓÇösource data 3.pdf]

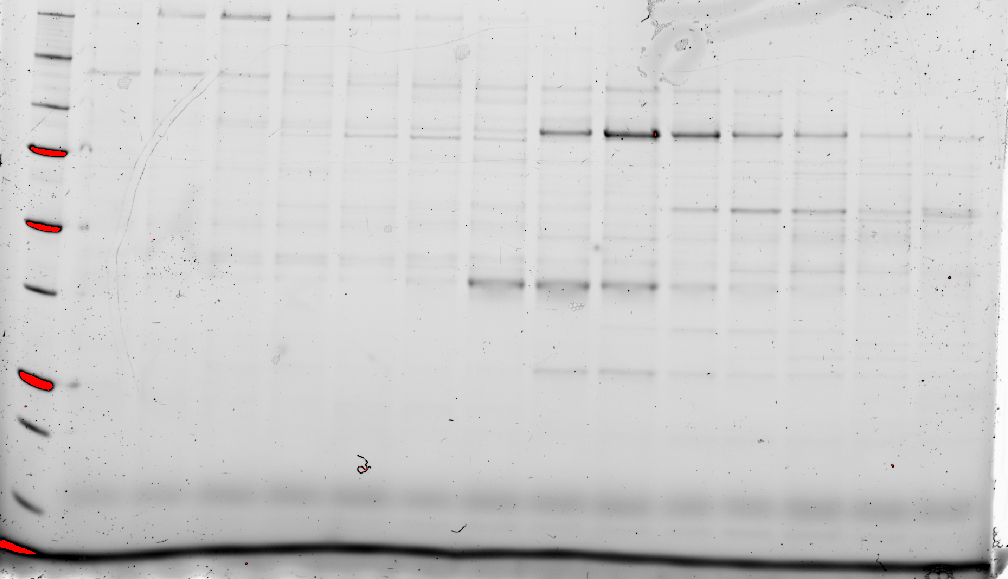

Supplement: Figure 4—source data 2. [file elife-89040-fig4-data2.zip › Figure 4ΓÇösource data 2/KLP-6(1-587)(D458A).tif]

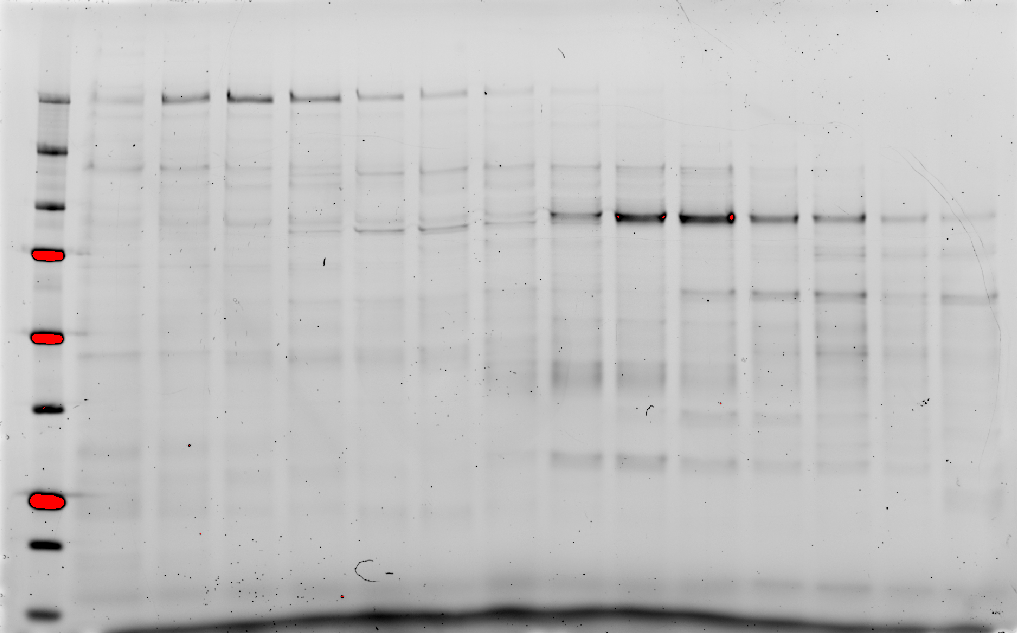

Supplement: Figure 4—source data 2. [file elife-89040-fig4-data2.zip › Figure 4ΓÇösource data 2/KLP-6(1-587).tif]

B

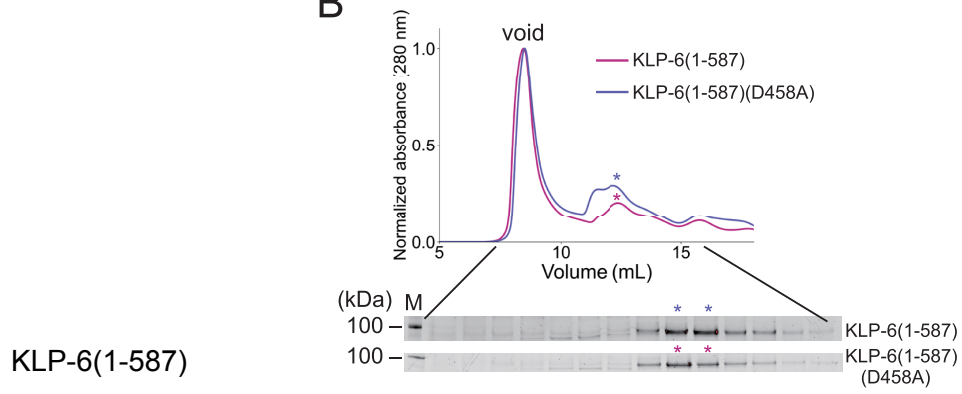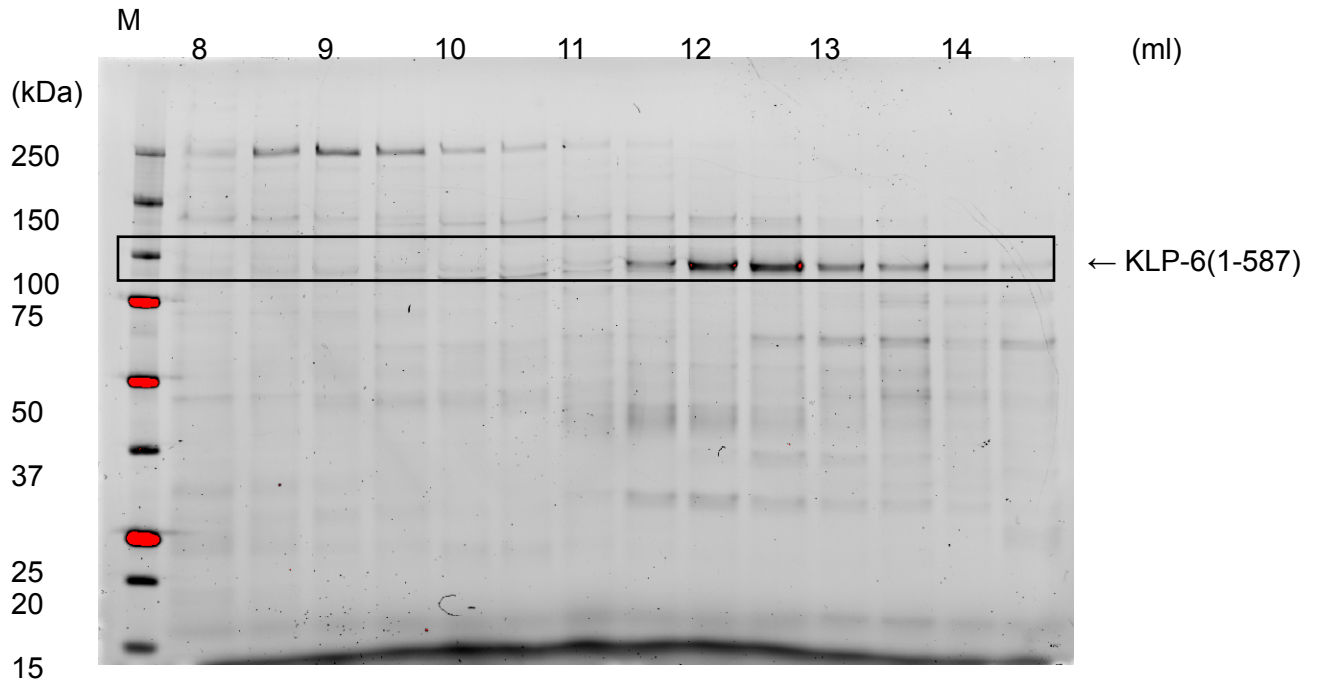

KLP-6(1-587)(D458A)

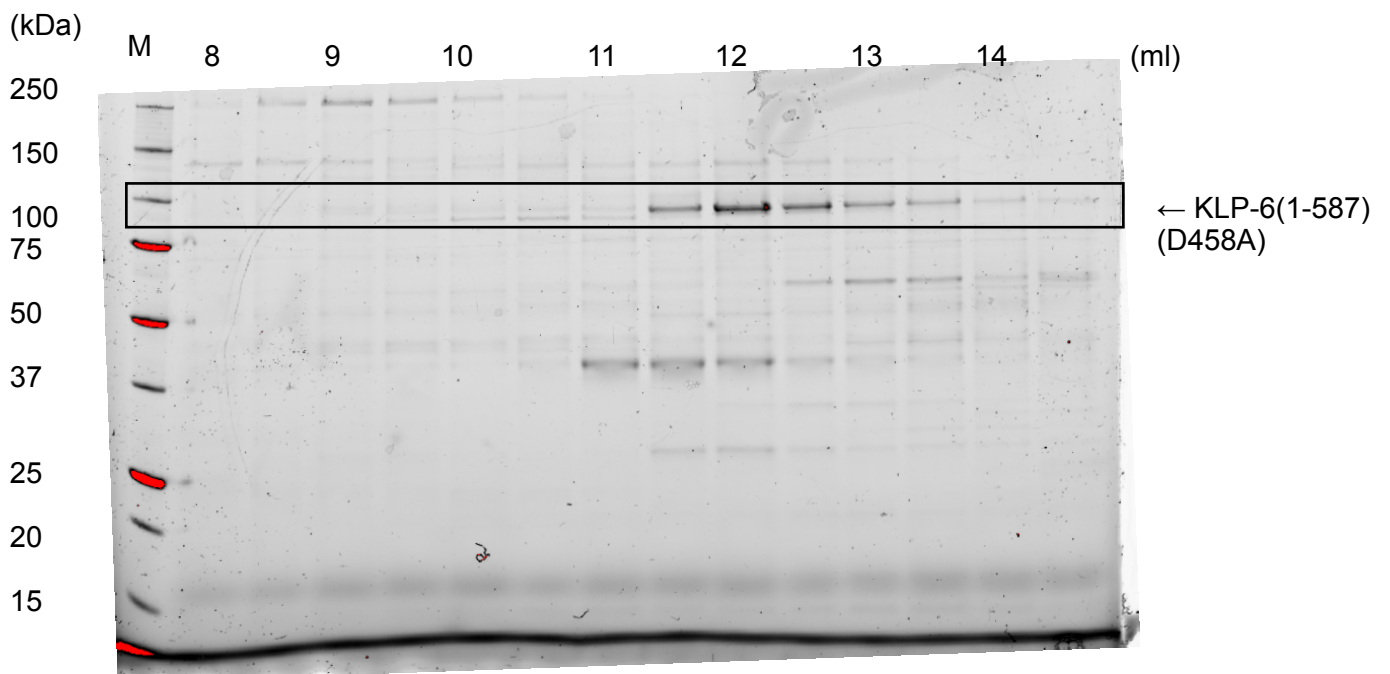

Supplement: Figure 4—source data 3. [file elife-89040-fig4-data3.zip › Figure 4ΓÇösource data 3/Figure 4ΓÇösource data 3.pdf]

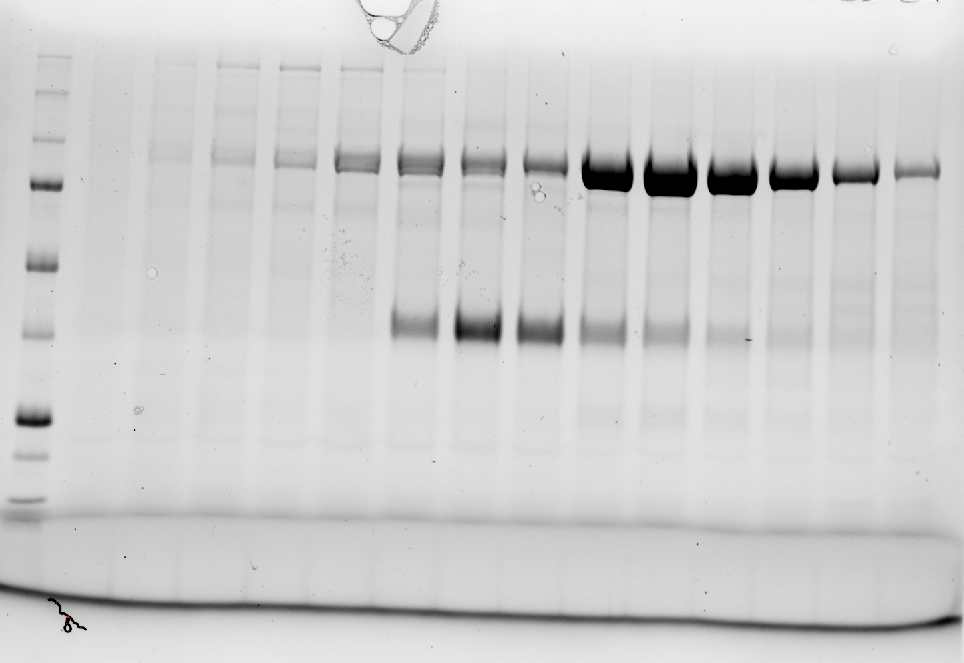

Supplement: Figure 5—source data 2. [file elife-89040-fig5-data2.zip › Figure 5ΓÇösource data 2/UNC-104(1-653).tif]

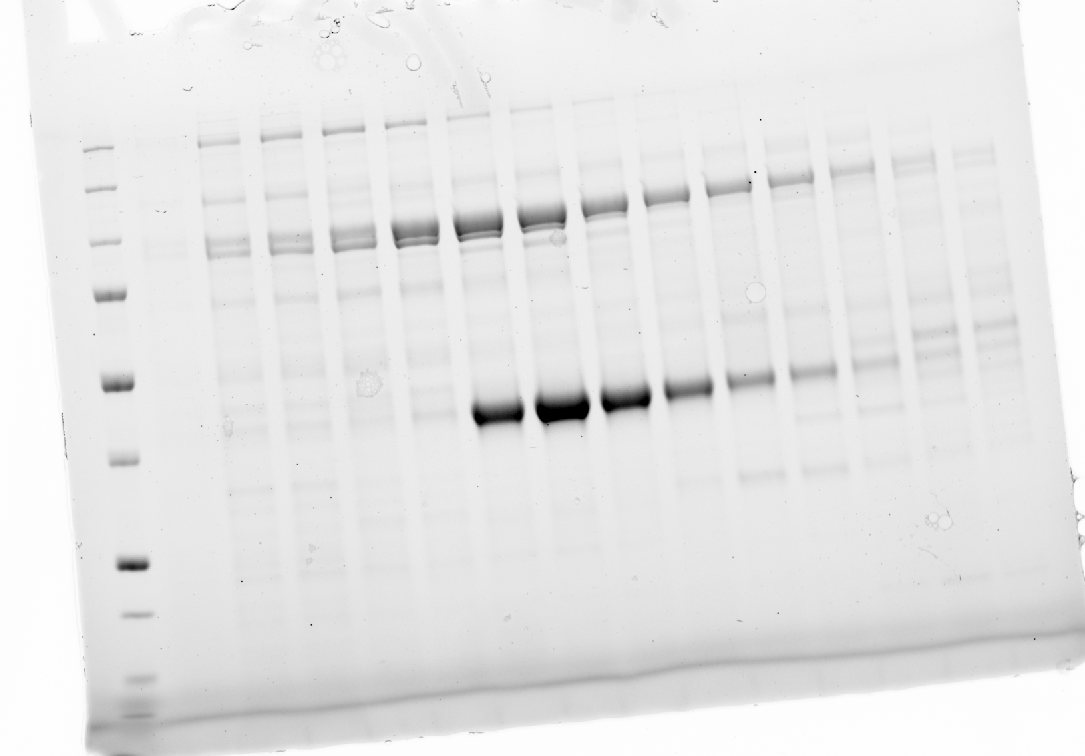

Supplement: Figure 5—source data 2. [file elife-89040-fig5-data2.zip › Figure 5ΓÇösource data 2/UNC-104(1-653)(E412K).tif]

**B**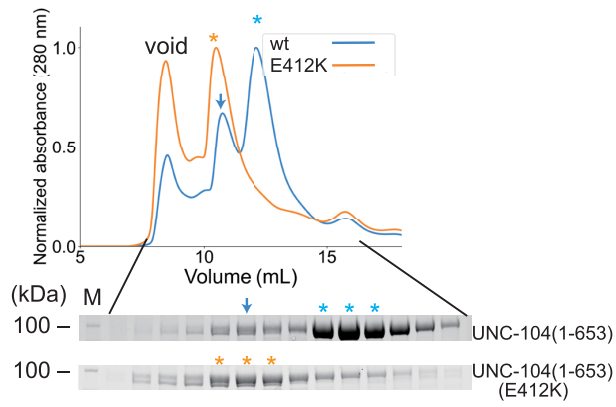

UNC-104(1-653)

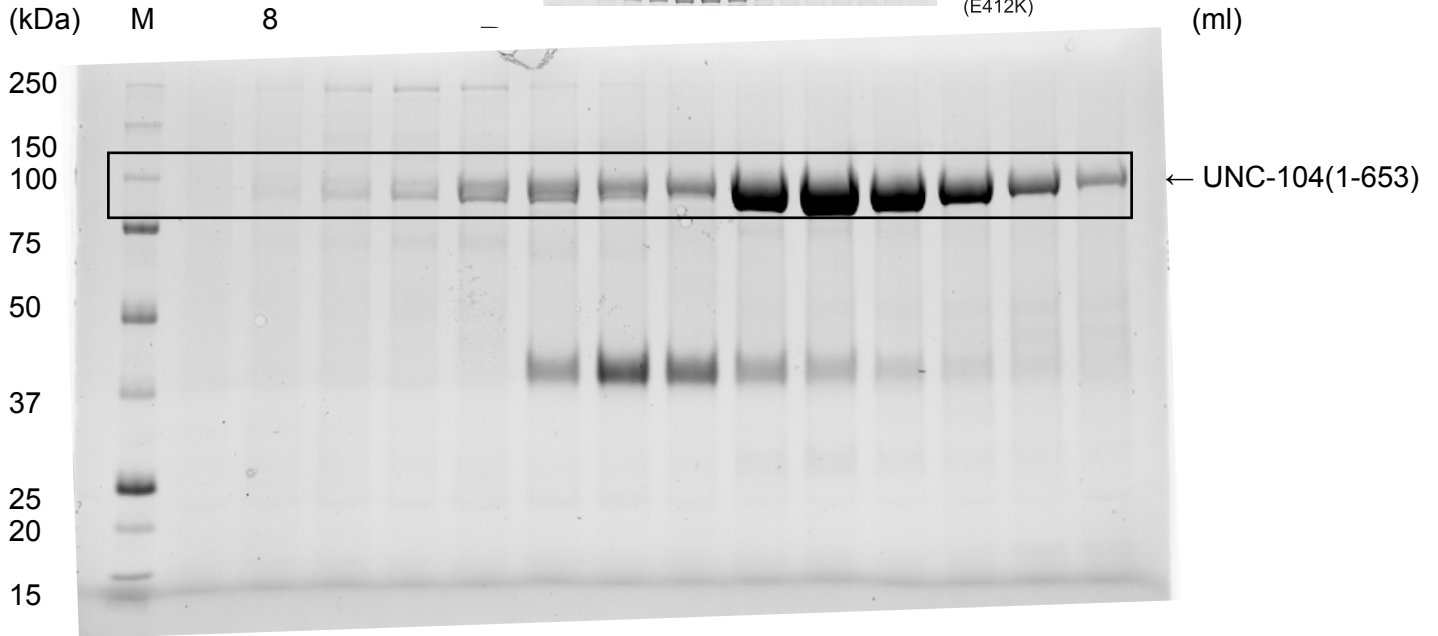

UNC-104(1-653)(E412K)

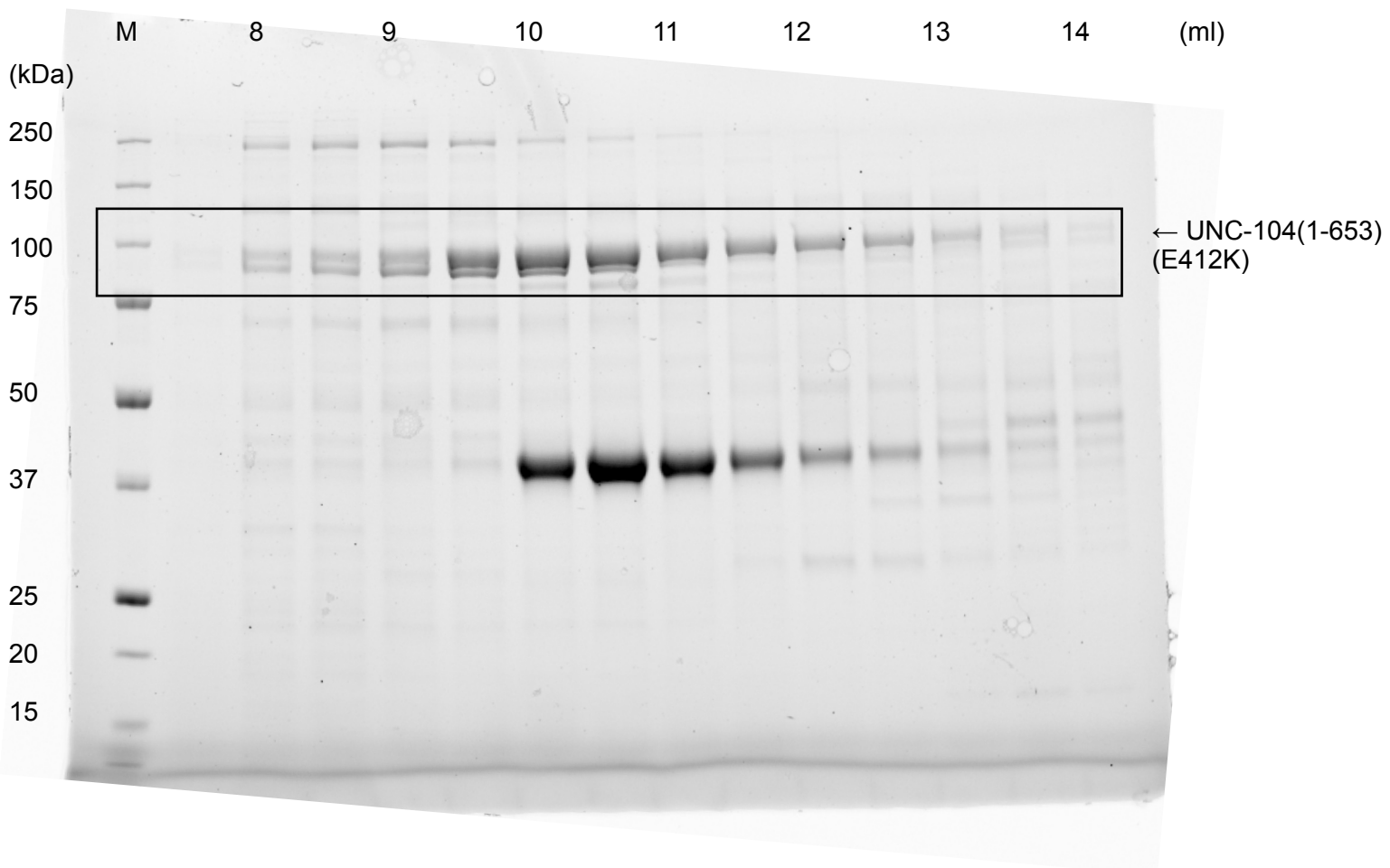

Supplement: Figure 5—source data 3. [file elife-89040-fig5-data3.zip › Figure 5ΓÇösource data 3/Figure 5ΓÇösource data 3.pdf]

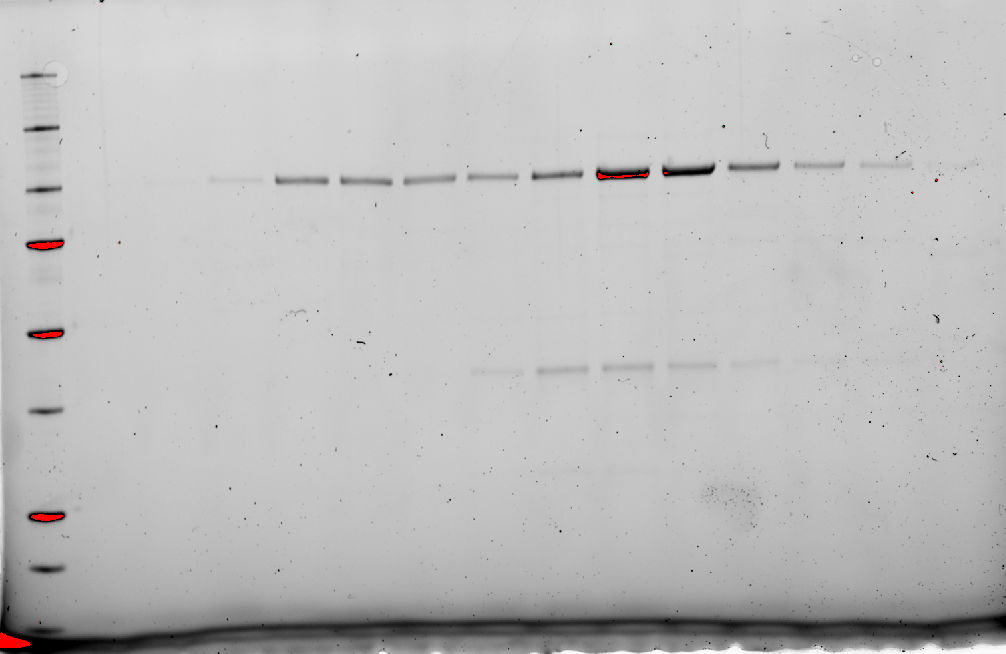

Supplement: Figure 5—figure supplement 1—source data 1. [file elife-89040-fig5-figsupp1-data1.zip › Figure 5-figure supplement 1ΓÇösource data 1/UNC-104(1-653)_72h_incubation.tif]

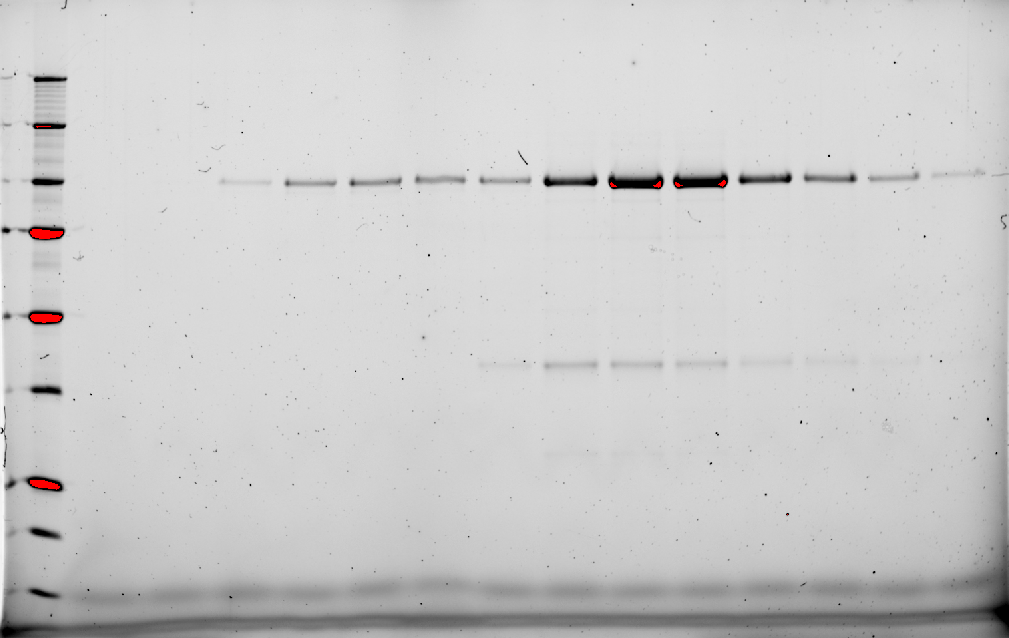

Supplement: Figure 5—figure supplement 1—source data 1. [file elife-89040-fig5-figsupp1-data1.zip › Figure 5-figure supplement 1ΓÇösource data 1/UNC-104(1-653)_No_incubation.tif]

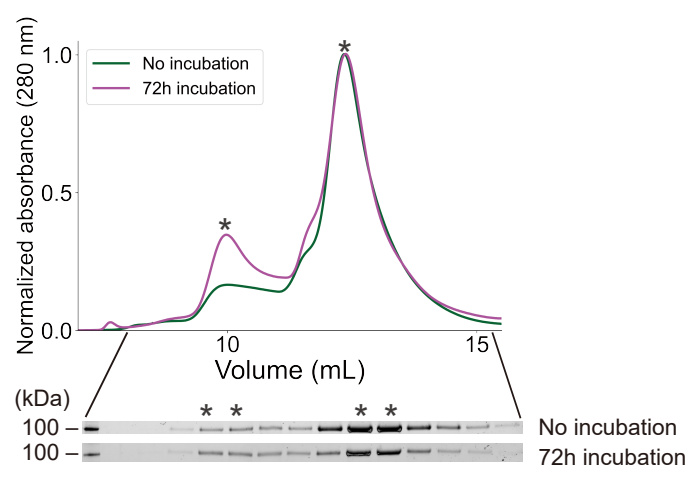

No incubation

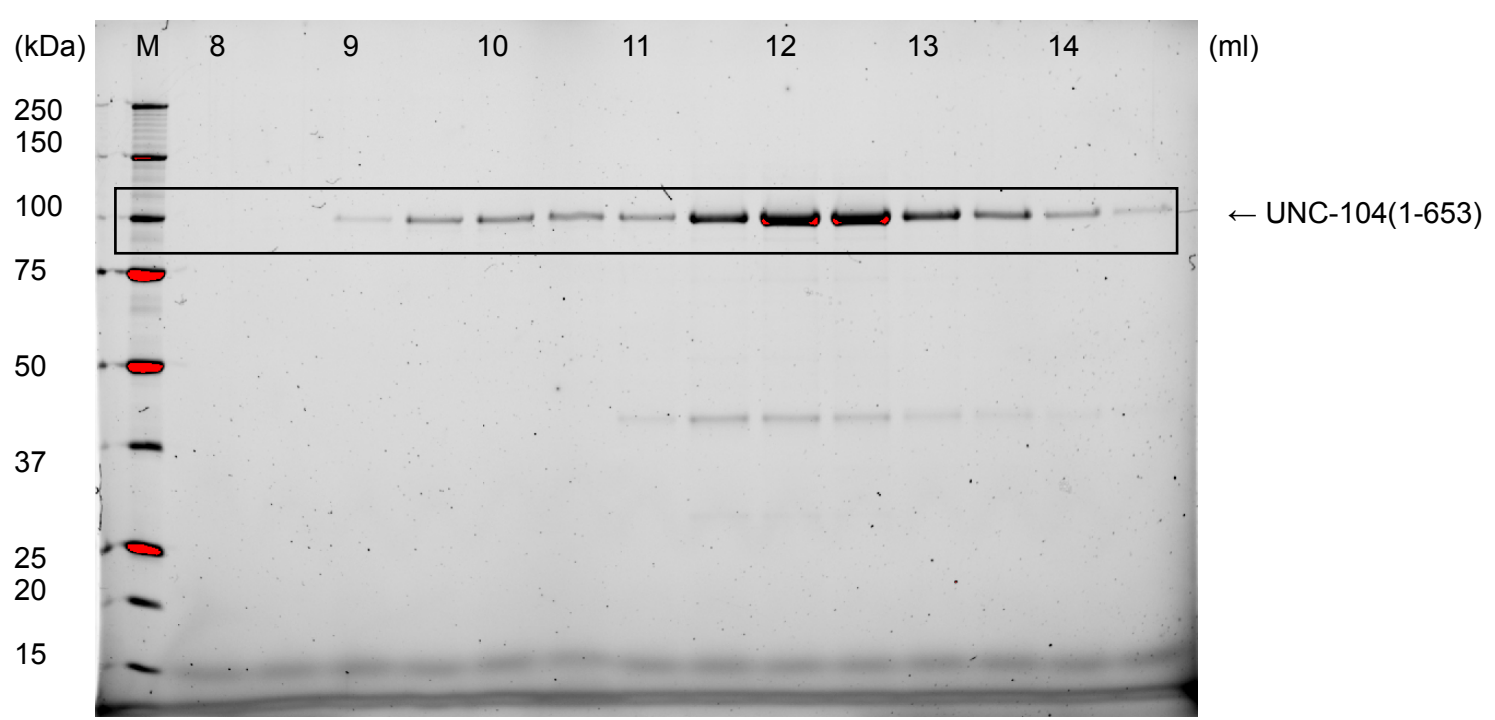

72h incubation

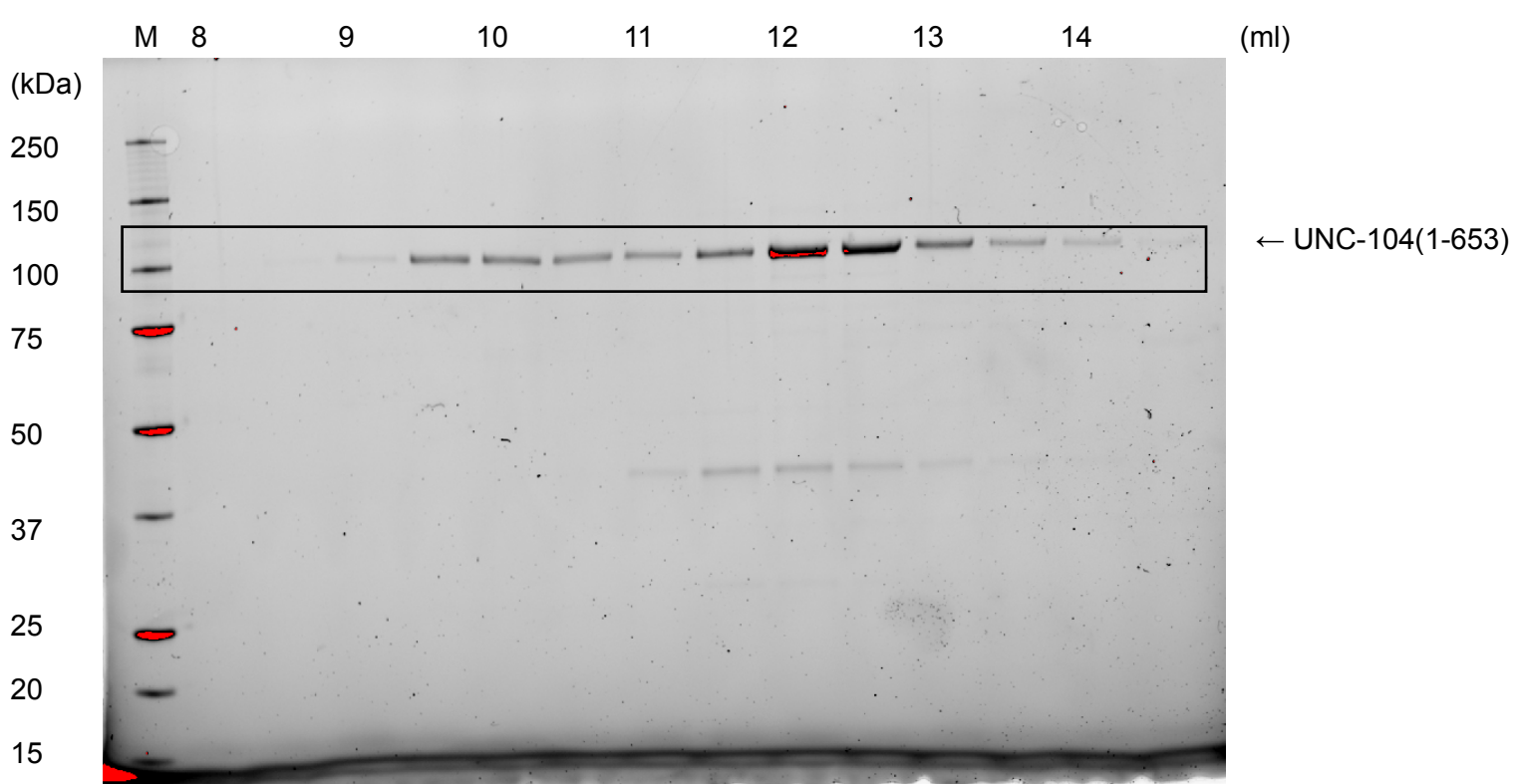

Supplement: Figure 5—figure supplement 1—source data 2. [file elife-89040-fig5-figsupp1-data2.zip › Figure 5-figure supplement 1ΓÇösource data 2/Figure 5-figure supplement 1ΓÇösource data 2.pdf]

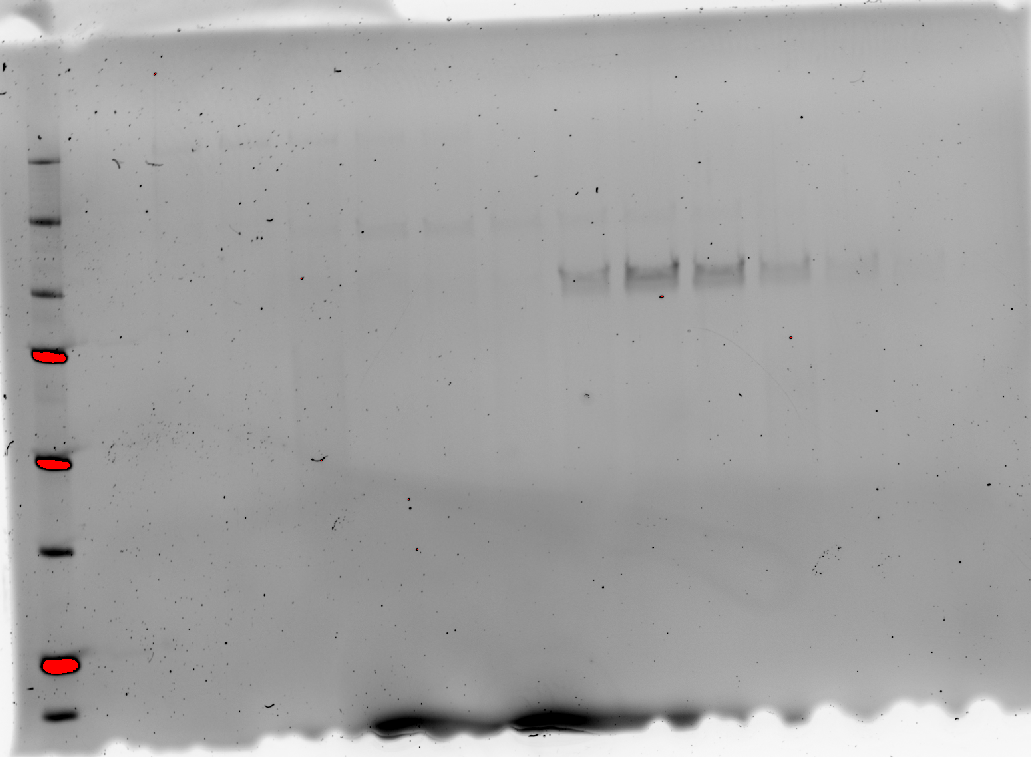

Supplement: Figure 5—figure supplement 3—source data 1. [file elife-89040-fig5-figsupp3-data1.zip › Figure 5-figure supplement3ΓÇösource data 1/KLP-6(1-587)(E409K).tif]

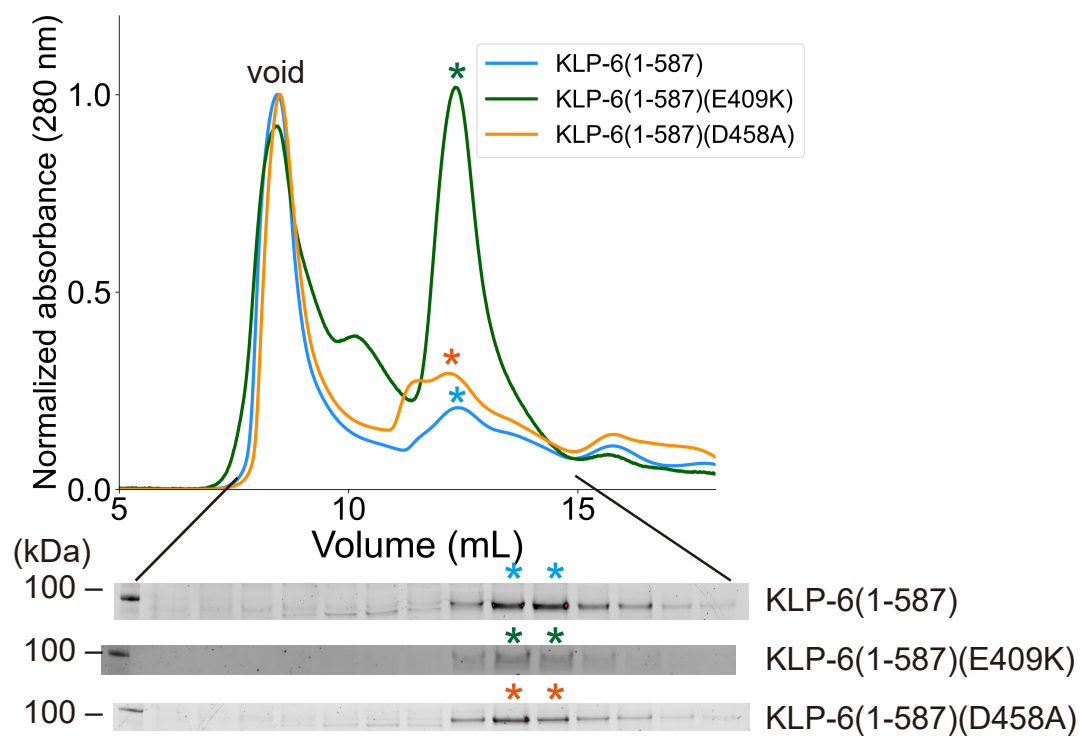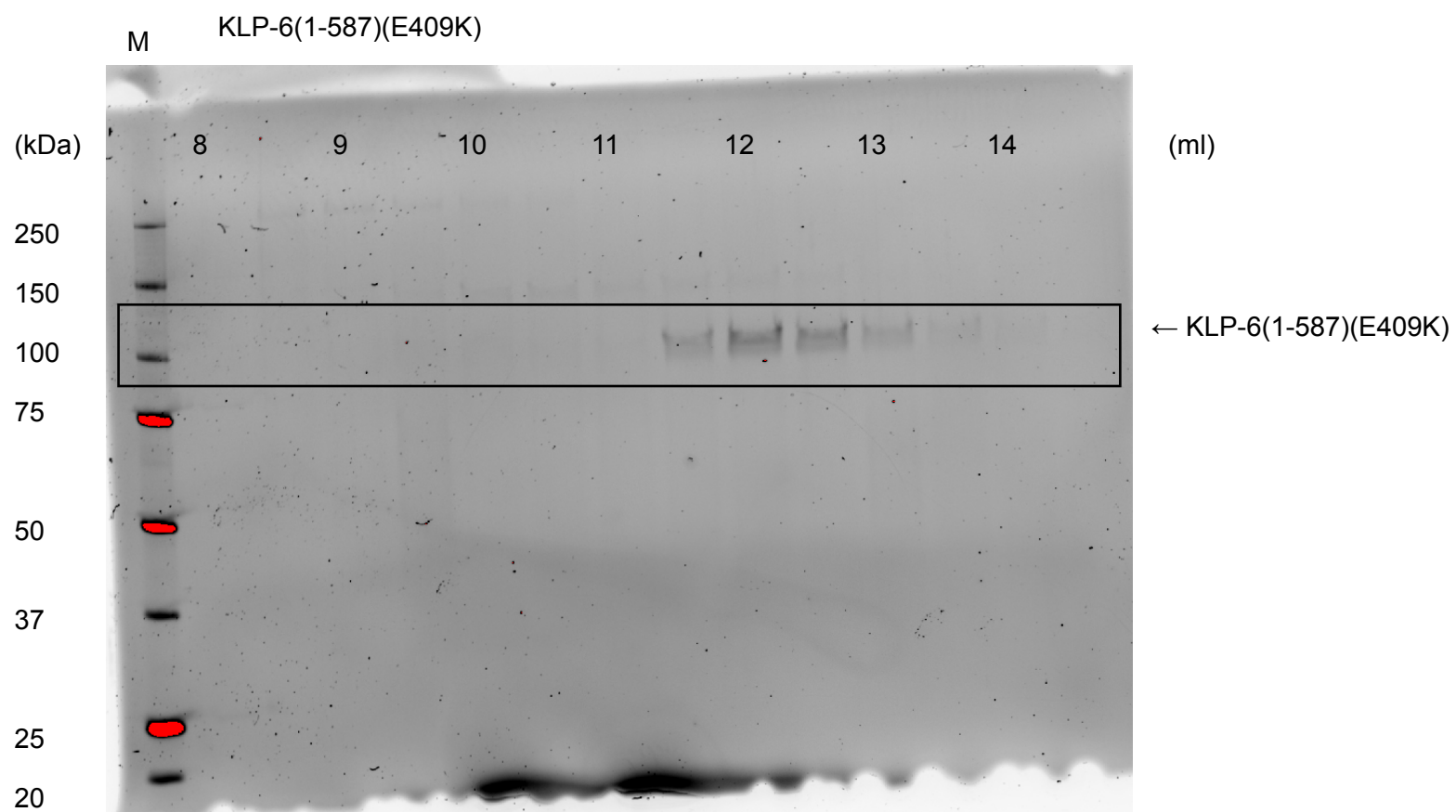

Supplement: Figure 5—figure supplement 3—source data 2. [file elife-89040-fig5-figsupp3-data2.zip › Figure 5-figure supplement3ΓÇösource data 2/Figure 5-figure supplement3ΓÇösource data 2.pdf]

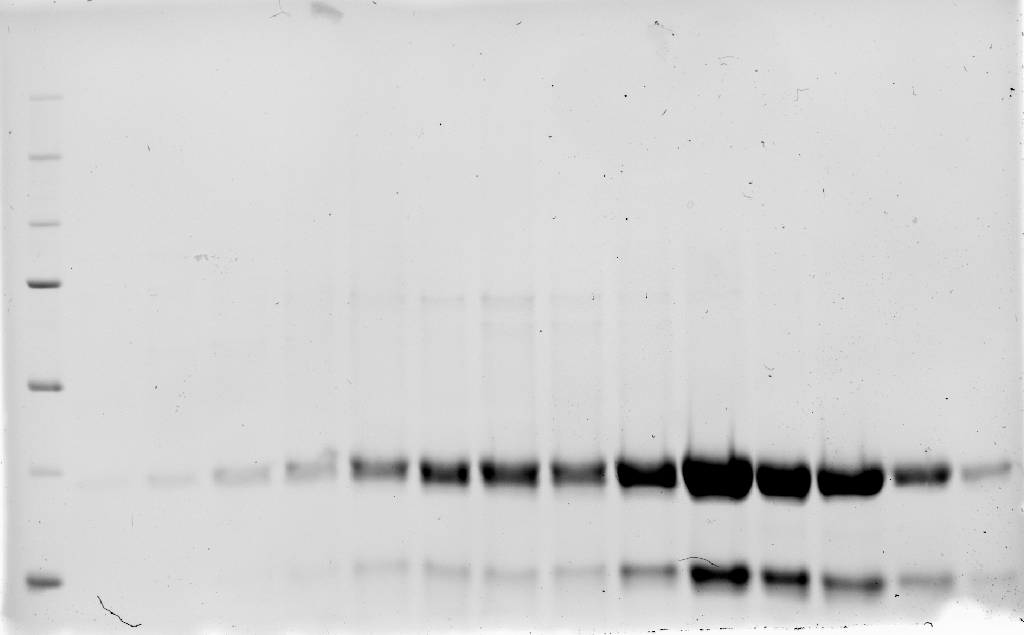

Supplement: Figure 6—source data 1. [file elife-89040-fig6-data1.zip › Figure 6ΓÇösource data 1/mSca.tif]

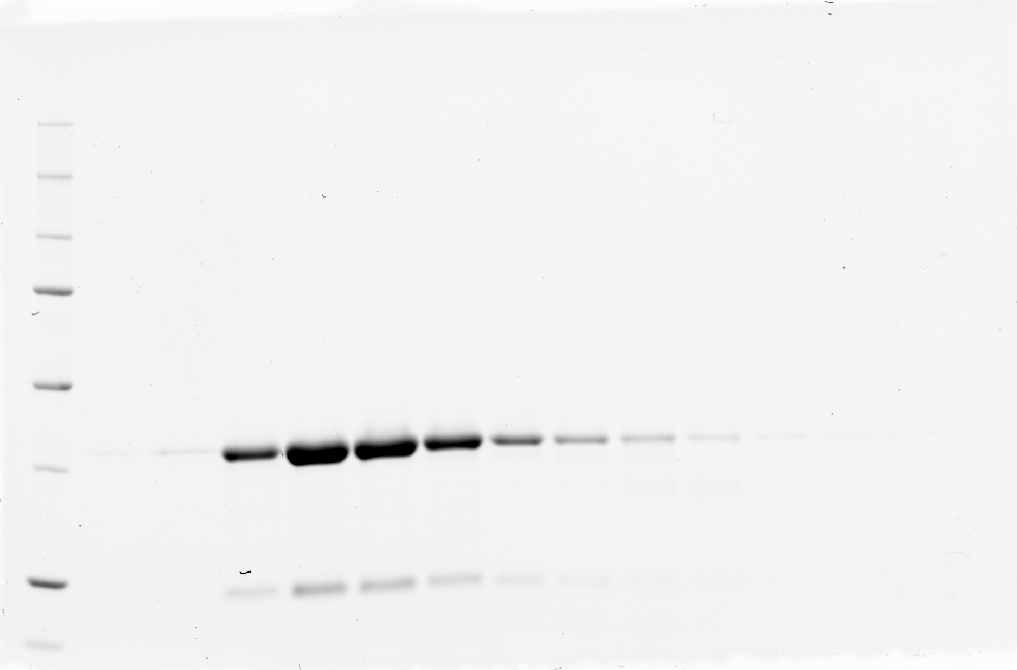

Supplement: Figure 6—source data 1. [file elife-89040-fig6-data1.zip › Figure 6ΓÇösource data 1/UNC-104CC2-mSca.tif]

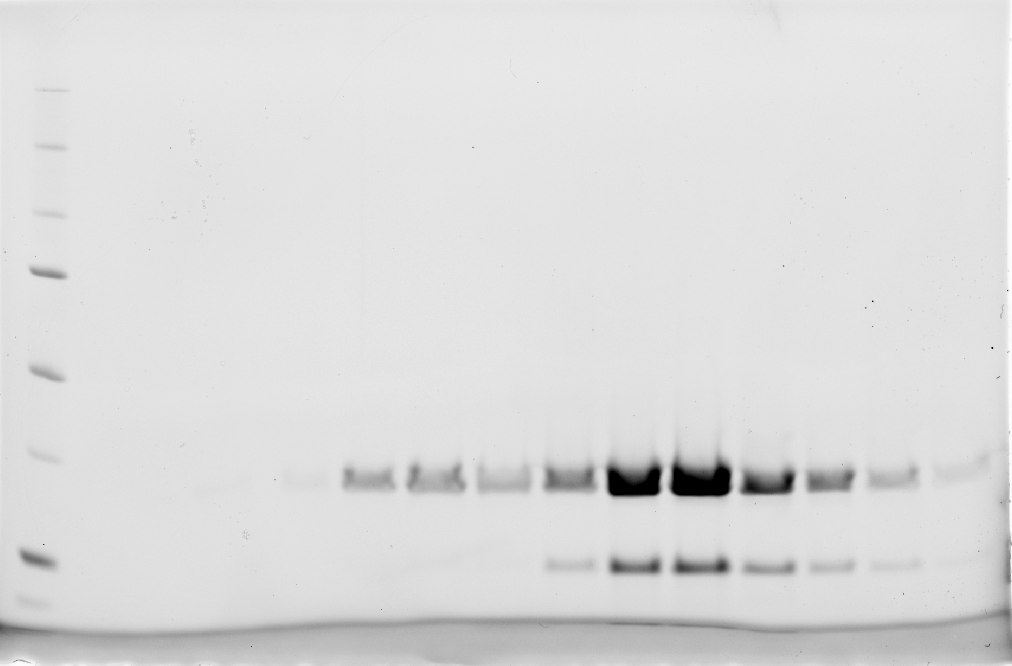

Supplement: Figure 6—source data 1. [file elife-89040-fig6-data1.zip › Figure 6ΓÇösource data 1/KLP-6CC2-mSca.tif]

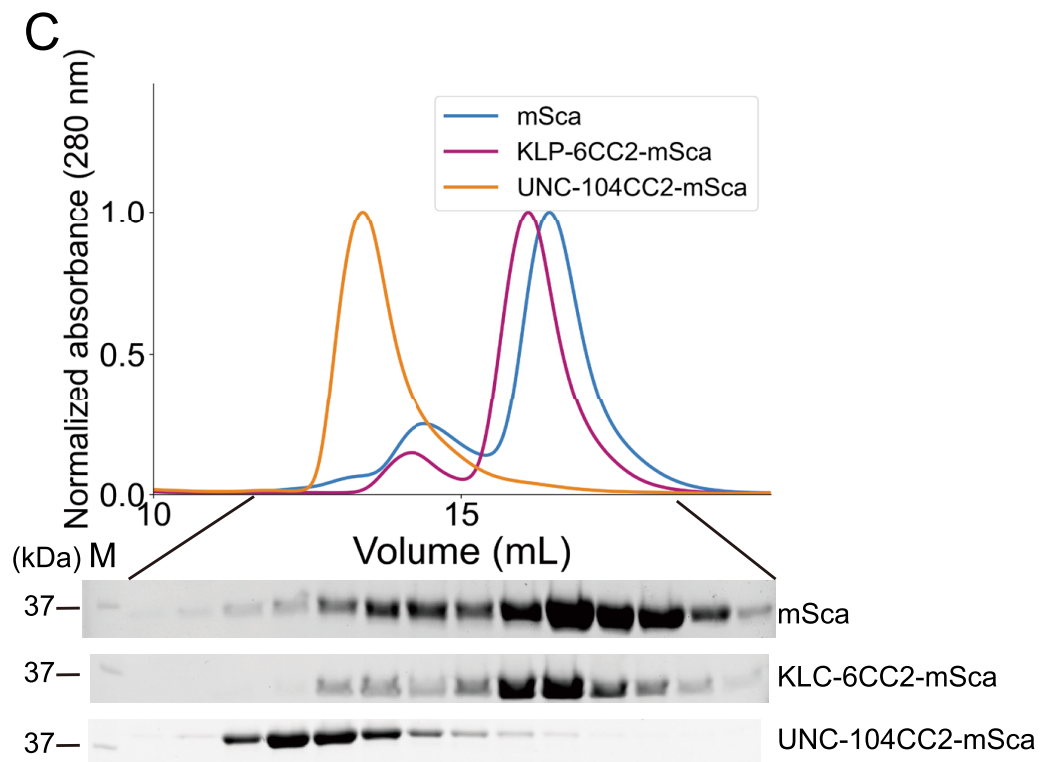

# mSca

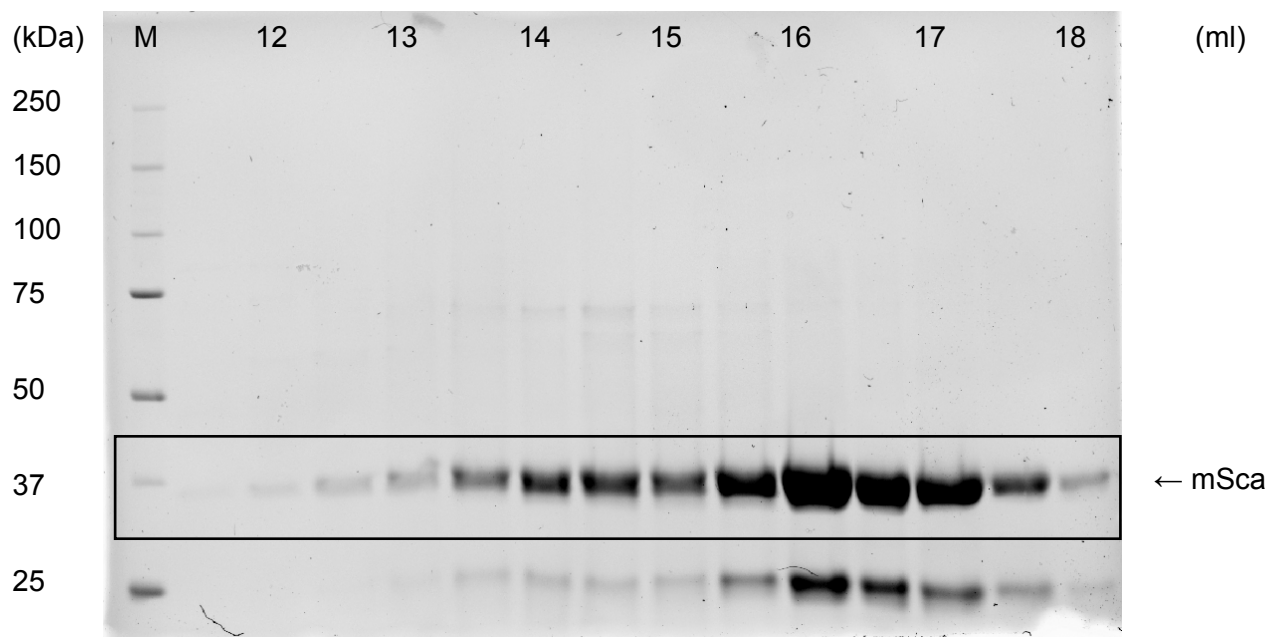

# KLP-6CC2-mSca

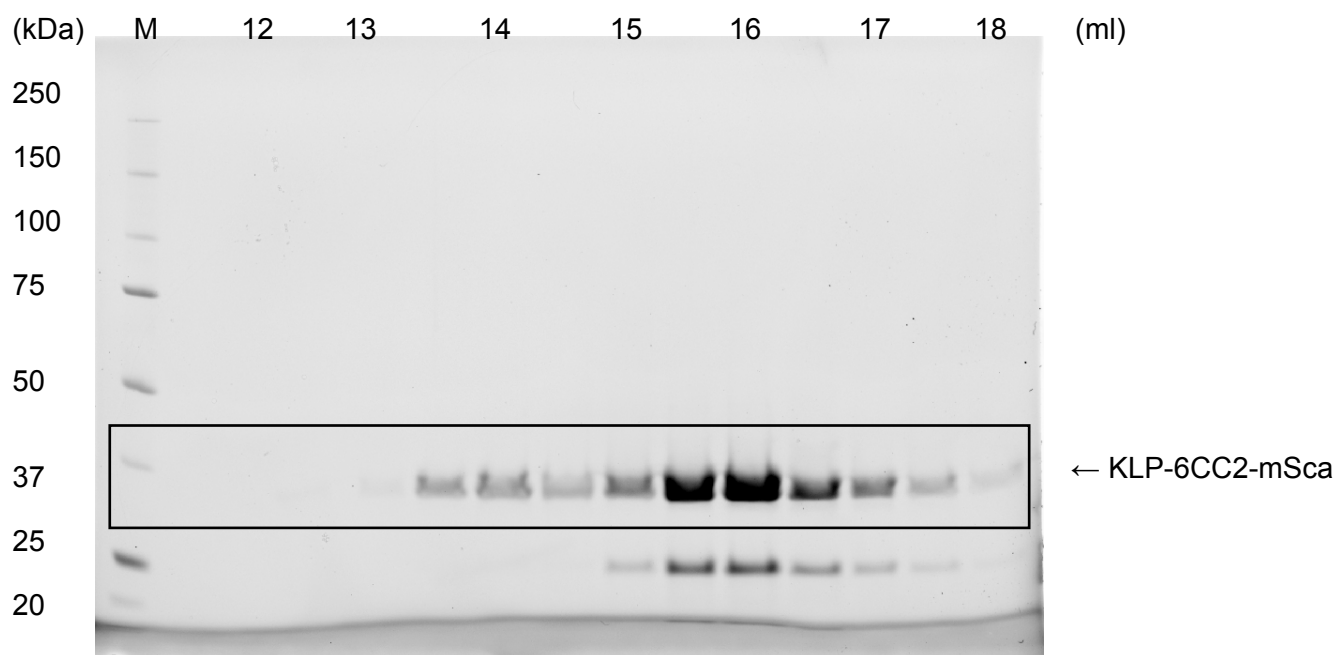

# UNC-104CC2-mSca

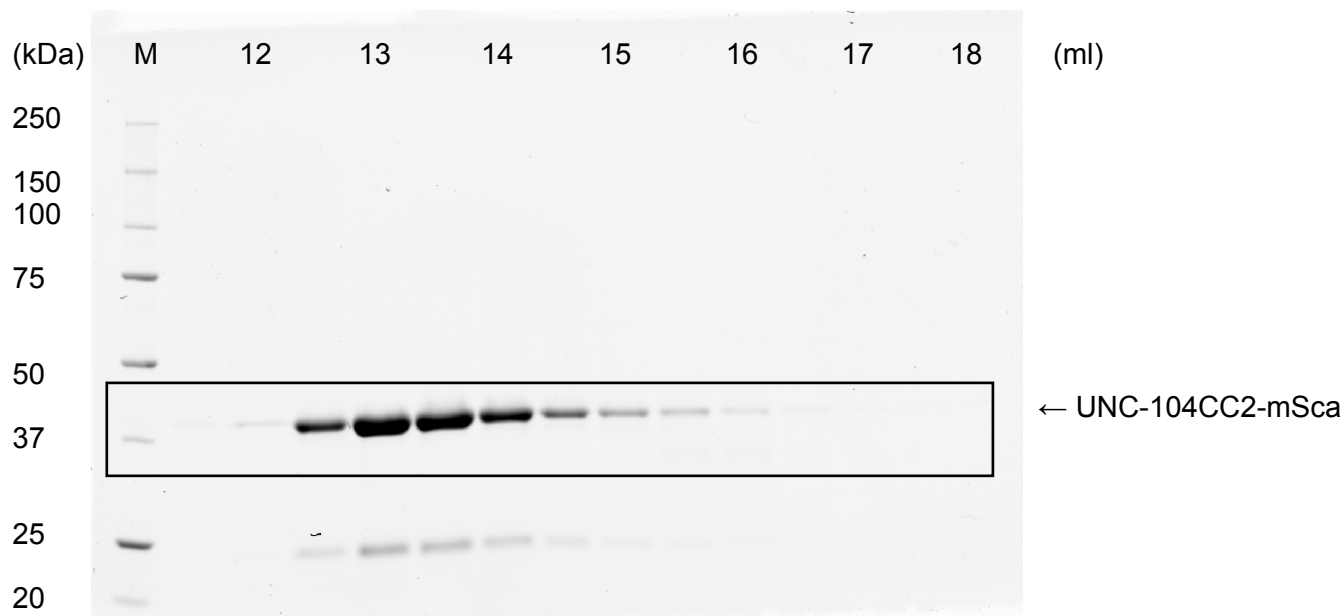

Supplement: Figure 6—source data 2. [file elife-89040-fig6-data2.zip › Figure 6ΓÇösource data 2/Figure 6ΓÇösource data 2.pdf]

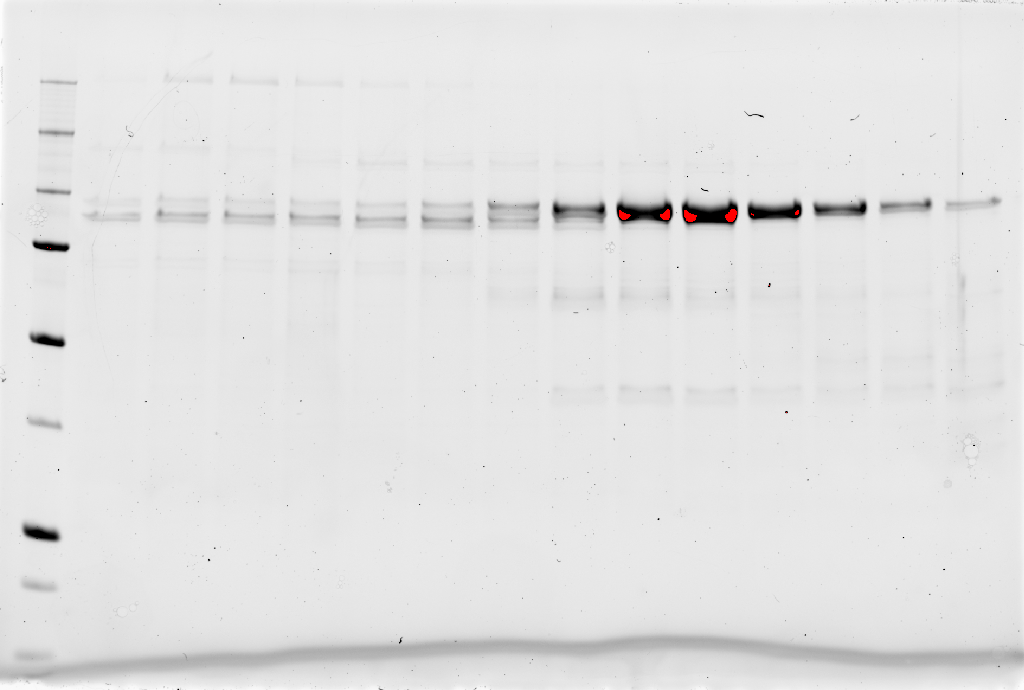

Supplement: Figure 7—source data 2. [file elife-89040-fig7-data2.zip › Figure 7ΓÇösource data 2/UNC-104(1-594).tif]

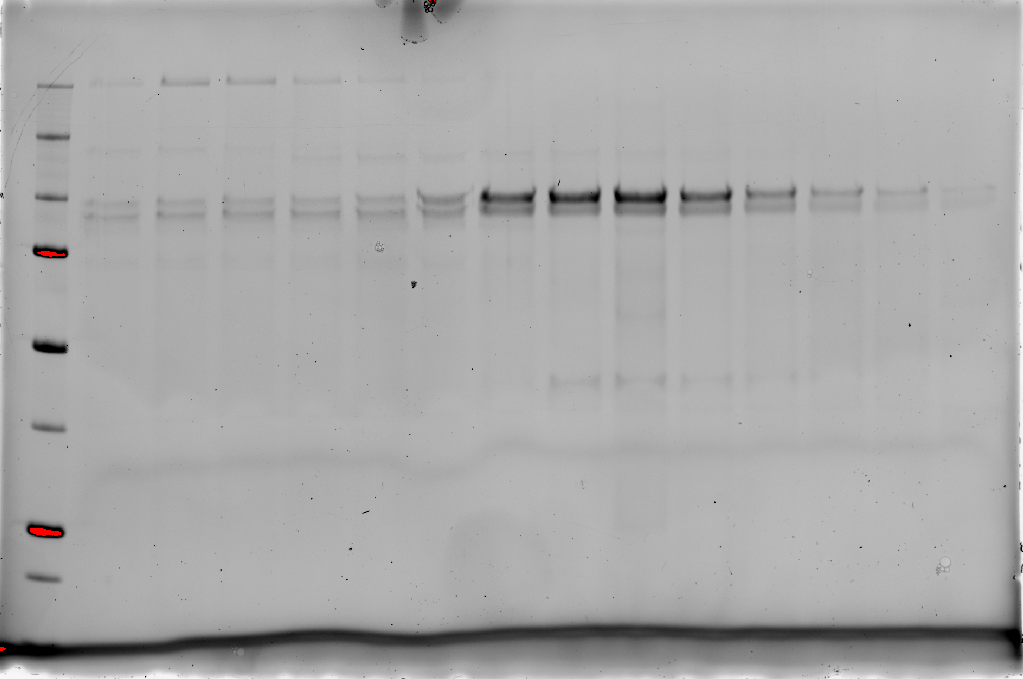

Supplement: Figure 7—source data 2. [file elife-89040-fig7-data2.zip › Figure 7ΓÇösource data 2/UNC-104(1-594)(E412K).tif]

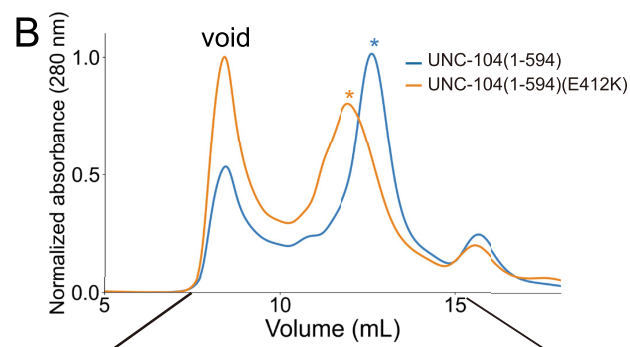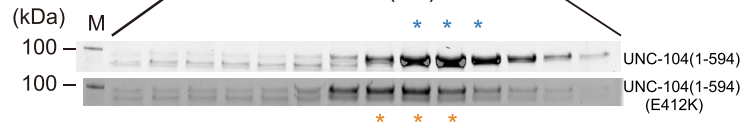

UNC-104(1-594)

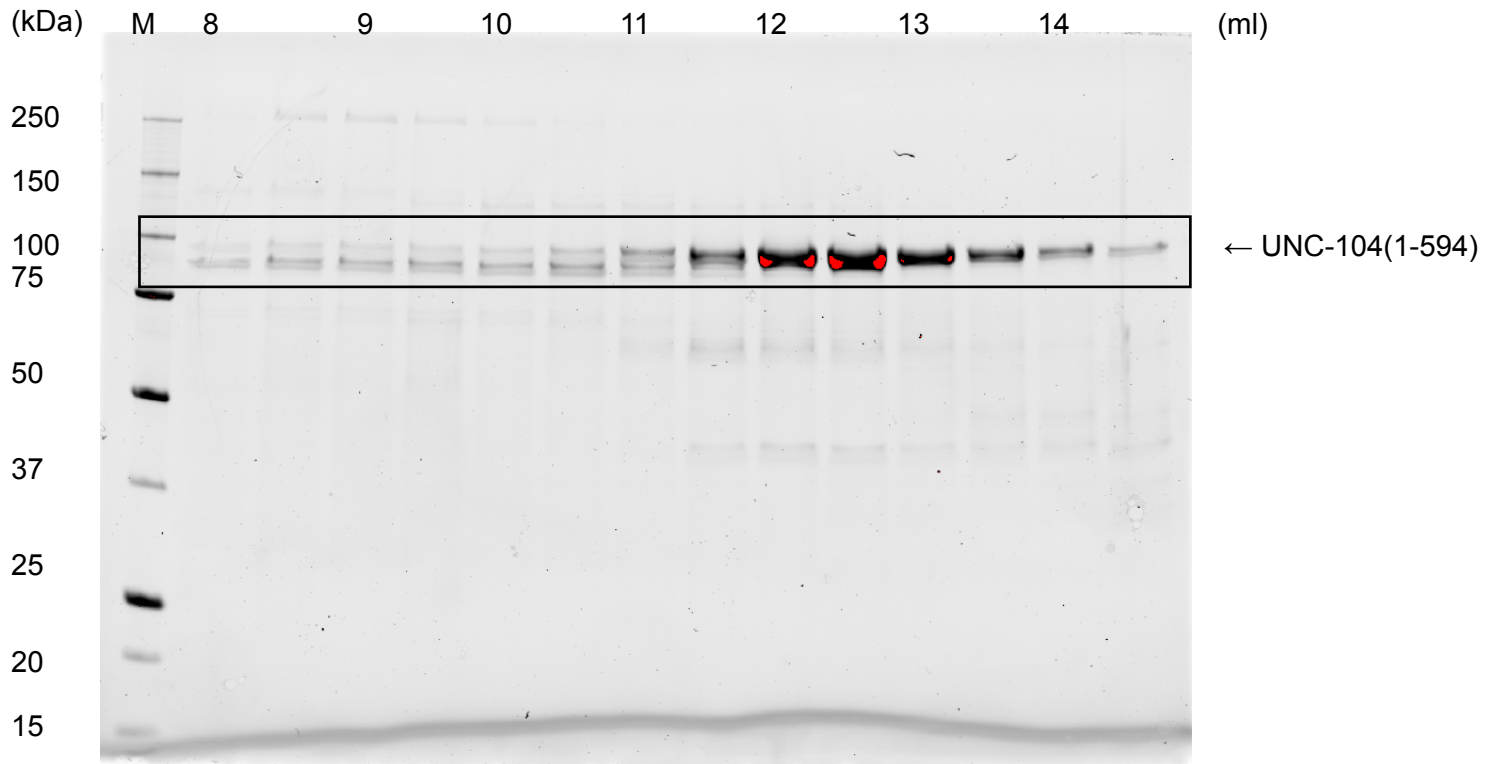

UNC-104(1-594)(E412K)

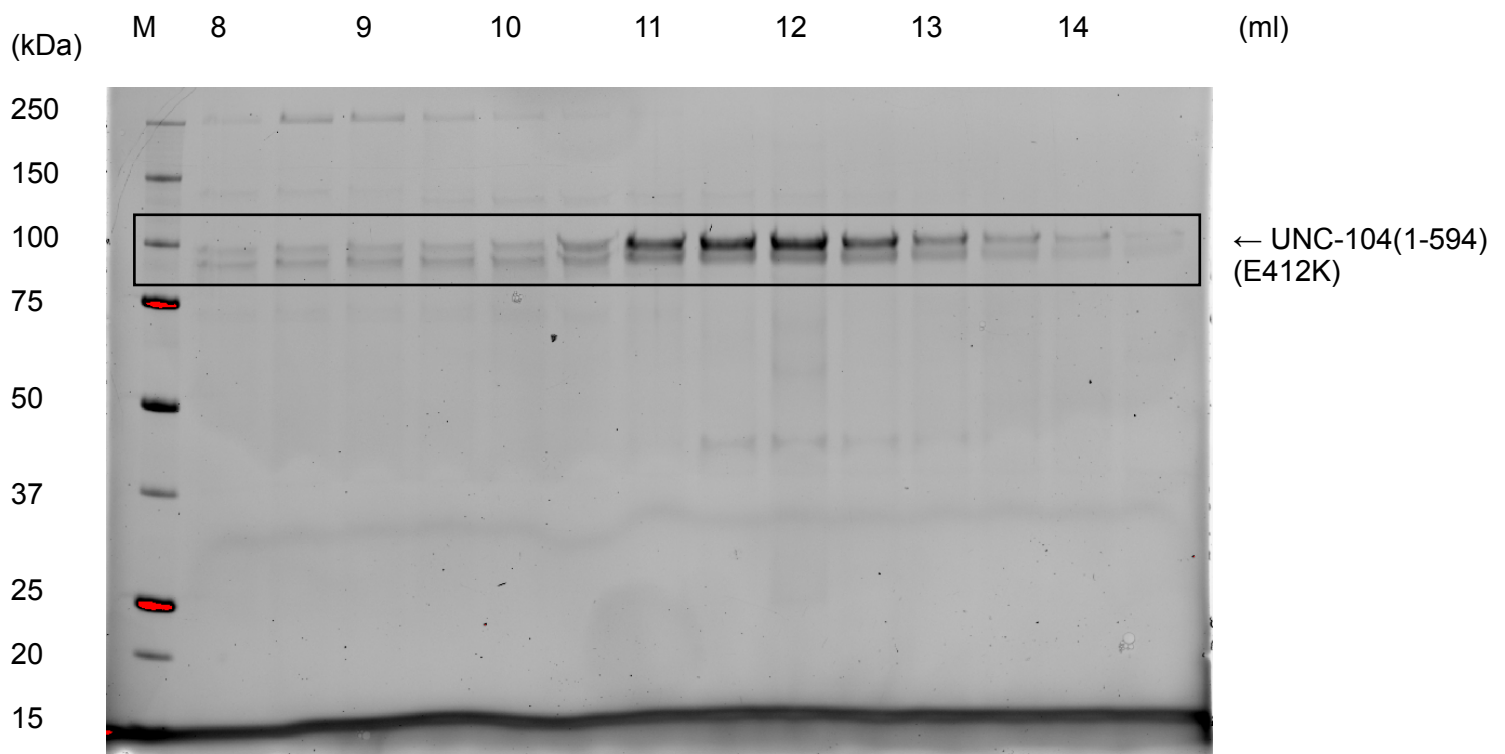

Supplement: Figure 7—source data 3. [file elife-89040-fig7-data3.zip › Figure 7ΓÇösource data 3/Figure 7ΓÇösource data 3.pdf]

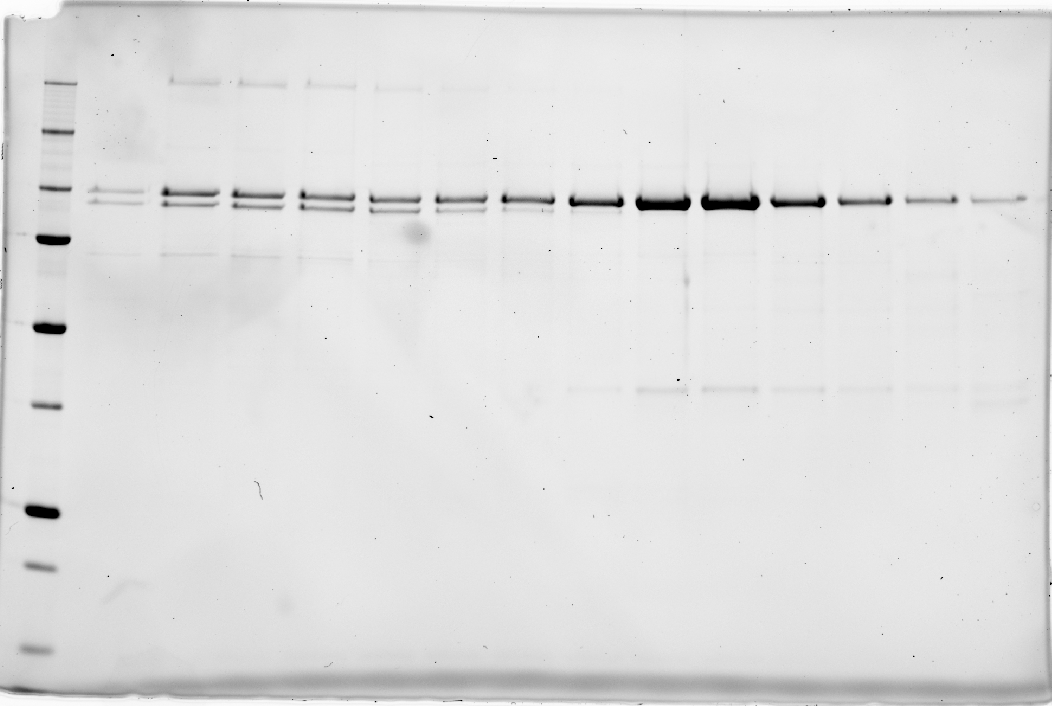

Supplement: Figure 7—figure supplement 1—source data 1. [file elife-89040-fig7-figsupp1-data1.zip › Figure 7-figure supplement1ΓÇösource data 1/UNC-104(1-653)(E412K,L640F).tif]

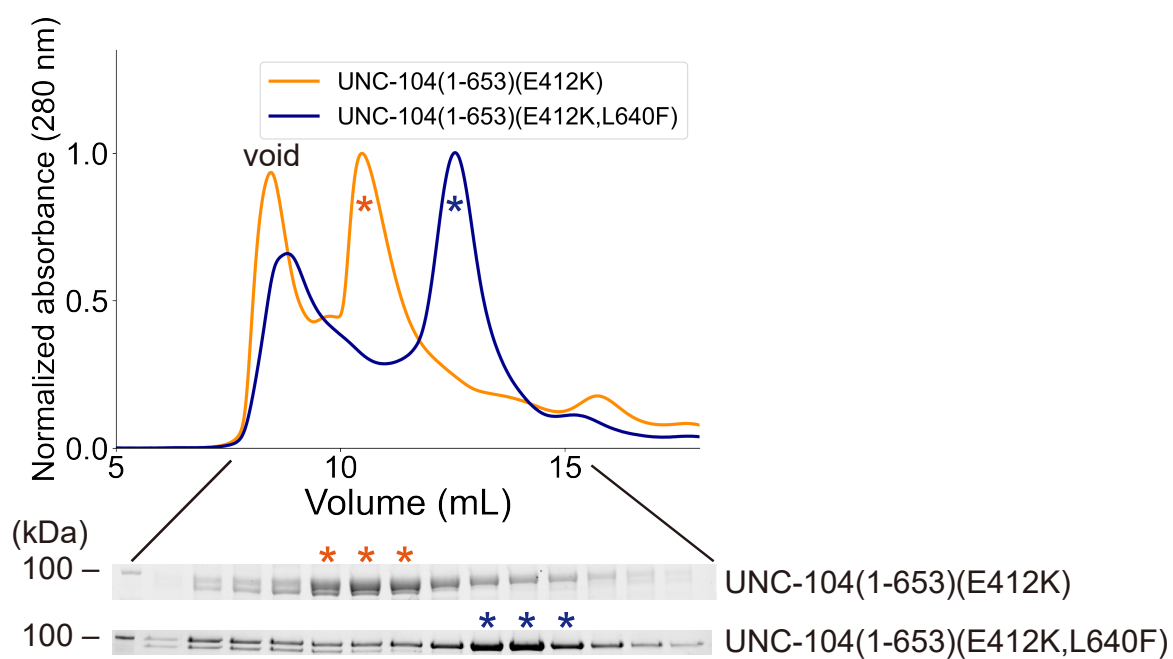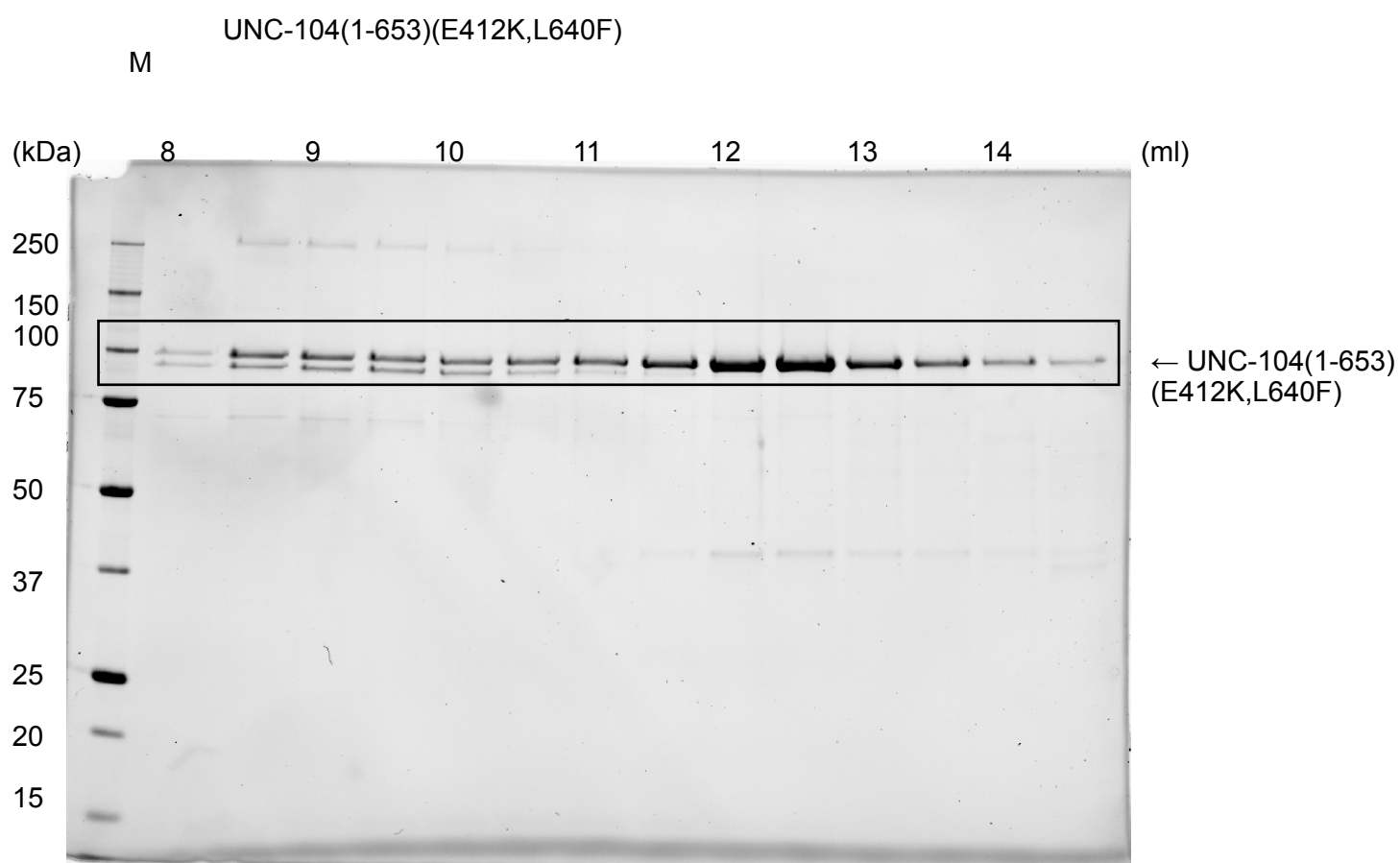

Supplement: Figure 7—figure supplement 1—source data 2. [file elife-89040-fig7-figsupp1-data2.zip › Figure 7-figure supplement1ΓÇösource data 2/Figure 7-figure supplement1ΓÇösource data 2.pdf]
